# Algorithmic improvements for discovery of germline copy number variants in next-generation sequencing data

**DOI:** 10.1186/s12859-022-04820-w

**Published:** 2022-07-19

**Authors:** Brendan O’Fallon, Jacob Durtschi, Ana Kellogg, Tracey Lewis, Devin Close, Hunter Best

**Affiliations:** ARUP Institute for Clinical and Experimental Pathology, Salt Lake City, UT USA

**Keywords:** Copy number variants (CNV), Next generation sequencing, Hidden Markov model, Whole exome sequencing

## Abstract

**Background:**

Copy number variants (CNVs) play a significant role in human heredity and disease. However, sensitive and specific characterization of germline CNVs from NGS data has remained challenging, particularly for hybridization-capture data in which read counts are the primary source of copy number information.

**Results:**

We describe two algorithmic adaptations that improve CNV detection accuracy in a Hidden Markov Model (HMM) context. First, we present a method for computing target- and copy number-specific emission distributions. Second, we demonstrate that the Pointwise Maximum a posteriori (PMAP) HMM decoding procedure yields improved sensitivity for small CNV calls compared to the more common Viterbi HMM decoder. We develop a prototype implementation, called Cobalt, and compare it to other CNV detection tools using sets of simulated and previously detected CNVs with sizes spanning a single exon to a full chromosome.

**Conclusions:**

In both the simulation and previously detected CNV studies Cobalt shows similar sensitivity but significantly fewer false positive detections compared to other callers. Overall sensitivity is 80–90% for deletion CNVs spanning 1–4 targets and 90–100% for larger deletion events, while sensitivity is somewhat lower for small duplication CNVs.

## Introduction

Accurate detection of germline copy number variants (CNVs) from hybridization-capture next generation sequencing (NGS) data remains a significant challenge. The sparse nature of targeted capture data typically precludes detecting CNVs via paired end mapping or split-read analysis, leaving read depth as the primary signal of DNA copy number. Read depth is subject to many confounding factors, including those that affect each region independently, such as GC or CpG content, as well as factors that create correlations in depths between regions, such as the presence of common CNVs or ‘batch effects’ produced by variable lab conditions. Elucidation of the true number of copies of an allele requires accounting for both sources of variability in additional to the stochastic nature of the fragment hybridization process.

Despite ongoing interest in the topic, several recent reviews and comparison papers have documented relatively low sensitivity, reproducibility, and positive predictive value (PPV) in read-depth based CNV detection methods. For instance, Hong et al. [1] examined four methods and found that PPV varied widely by data set and ranged from approximately 10–80% for deletions and was uniformly below 60% for duplications. Comparing three methods on whole-exome sequencing (WES) data, Yao et al. [2], found similarly low PPV and sensitivity. Tan et al. [3] also noted high Mendelian error rates in a set of exome trios and poor concordance of CNV calls between callers. Contrasting perspectives can be found in de Ligt [4] and Yamamoto et al. [5], who found relatively high sensitivity and specificity in larger, multi-gene CNVs using the Krumm [6] and Fromer [7] methods.

Many methods have been proposed to estimate copy-number status from NGS read depth data, but most involve two primary tasks. First, raw read depths are ‘normalized’ to minimize variability and remove depth artifacts from non-CNV sources. Second, these normalized depths must undergo a multiclass segmentation procedure to assign distinct copy numbers to each targeted region.

Read depth normalization procedures involve comparing the raw read depth at a particular base or region to that from a set of control samples. The most straightforward of these techniques involve examining the ratio of read depth in the query sample relative to the controls, often after correcting for GC-content (e.g. [8–10]). When a large panel of control samples is unavailable, some authors have employed LOWESS smoothing to reduce stochastic noise (e.g. [11]). Such procedures reduce the target-to-target variability present in hybridization-capture NGS data, but may fail to eliminate variability due to the correlational structure of depths across targets. To ameliorate such effects, Krumm et al. [6] and Fromer et al. [7] introduced similar techniques that involve decomposing the matrix of depths across control samples using singular value decomposition (SVD). The approach removes a configurable fraction of the variation present under the assumption that the most prevalent sources of variation are unlikely to be due to CNVs. Jiang et al. [12] present an alternative method based on Poisson latent factors that explicitly models GC content in addition to systematic biases.

Several techniques have been used for segmenting normalized depths into CNV calls. Some methods adopt a thresholding algorithm whereby any target with a normalized depth exceeding a threshold value is assigned to a CNV state [6, 8, 9]. Other methods employ a Hidden Markov Model (HMM). While HMMs naturally incorporate the stochastic variability in read depths, they are often parameter-rich, requiring choices for the transition matrix as well as means, variances, and possibly other parameters for the emission distributions associated with every target. Because many NGS studies examine ten of thousands of targets, even small errors in fitting the distributions may result in a large number of false positive calls and low positive predictive value (PPV). Fromer et al. [7] utilize an HMM in which emission distributions are fixed for all targets and must be specified by the user, while Love et al. [13] and Packer et al. [10] construct an HMM whose parameters are estimated in a maximum likelihood procedure. In all cases the Viterbi algorithm is employed to identify the most likely copy number states at each target. Jiang et al. [12] develop a novel procedure that uses Circular Binary Segmentation (CBS, [14]) to segment likelihood ratios from a Poisson model. In the context of single-cell sequencing, Zhang et al. [11] modeled read depths at each site with a negative binomial distribution, and used a numerical optimization method to identify most likely copy number states while maximizing overall smoothness and sparsity.

Here, we introduce a method that combines ideas from Love et al. [13], Packer et al. [10] and Fromer et al. [7]. Similar to the Fromer et al. [7] work, we use SVD to identify and remove common sources of variation from raw read depth data. Our approach combines two innovations. First, we describe a new method of estimating target- and state-specific emission distribution parameters. Second, in contrast to other HMM-based methods, we decode the HMM using the pointwise maximum a posteriori (PMAP) algorithm [15], instead of the more typical Viterbi algorithm, in an effort to maximize the number of correctly called targets. We compare Cobalt to six other detection tools using both simulated and orthogonally detected CNVs of all sizes. We also demonstrate performance improvements and parallelization opportunities resulting from partitioning of the targets into independent groups.

## Implementation

### Cobalt

**Fig. 1:**
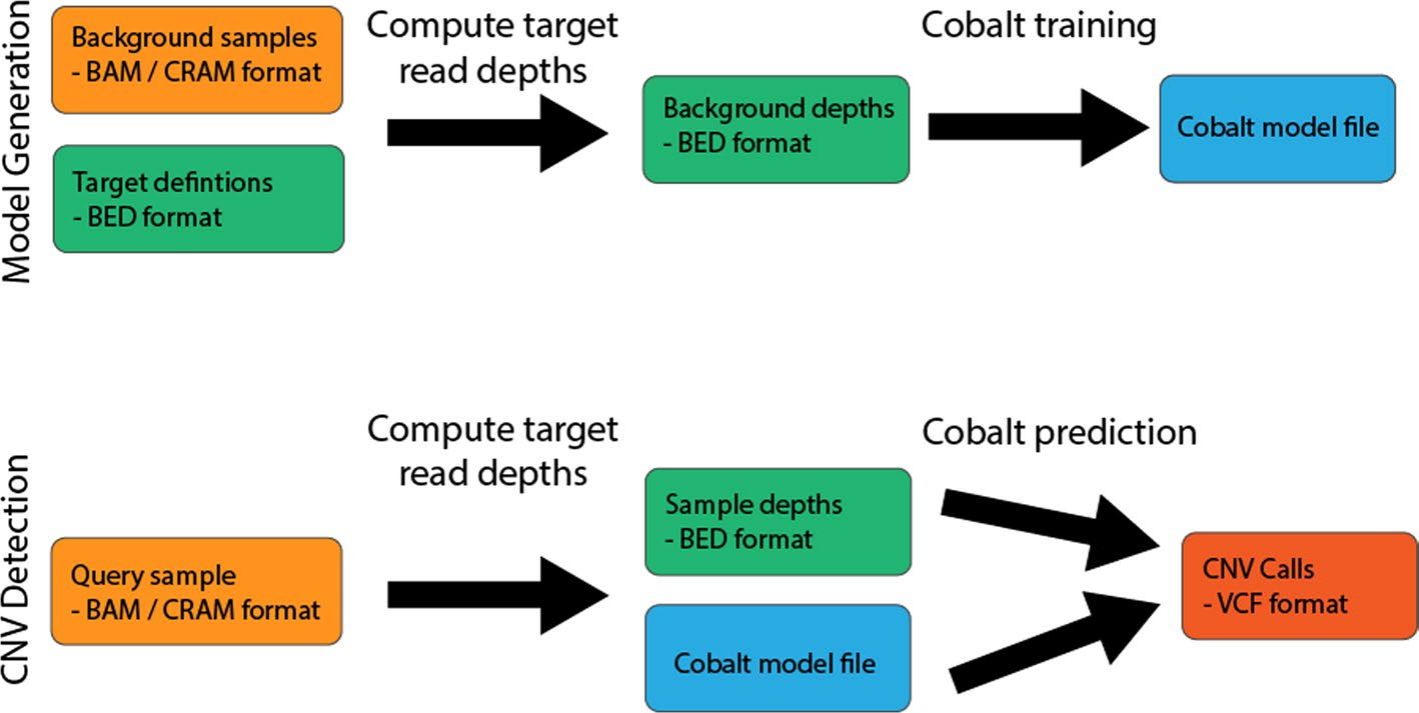
Overview of steps involved in the training (top) and prediction (bottom) phases for CNV detection with Cobalt

Our algorithm operates in two phases, a ‘training’ phase where data from control samples is used to create a reusable model, and a ‘prediction’ phase where the model is used to detect CNVs in a single query sample. An overview of the phases is shown in Fig. 1.

#### Training

During training, read count depths from multiple samples are used to generate a collection of parameters referred to as a ‘model’. The samples used for training are termed the ‘background’ or ‘control’ samples. Read count data must be provided in a BED-formatted file, with rows corresponding to targets and columns containing read counts for the samples. Targets do not need to be of similar sizes, but should not be overlapping. Our implementation does not assume any particular method of obtaining read counts - both mean target read depth or the total number of reads overlapping a target region are suitable. Under typical use each target corresponds to a single hybridization probe location, although special considerations should be taken if there are multiple overlapping probe locations.

Read depth data are converted into a $n \times p$ matrix *D*, with *n* targets and *p* samples. We then compute *P* such that

1 $$\begin{aligned} P = ln(D+1) \end{aligned}$$

After centering each column of *P* about the column median to produce *R*, we then compute the *k* right singular vectors of $R^{T}$. *k* is computed as the minimum value such that the proportion of variance explained by singular vectors 0..*k* is at least *v*, which by default is 0.90 but can be changed by the user. In “Target partitioning” we discuss two strategies for minimizing computational costs for large *n*. Setting $V_k$ to be the column matrix of the singular vectors, we then compute

2 $$\begin{aligned} T = R - RV_k^{T}V_k \end{aligned}$$

The centering procedure removes target-specific biases due to, for instance, genomic GC or CpG content, while the SVD procedure removes across-target correlations due to CNVs present in the samples or ‘batch effects’ that induce similar changes across sets of targets.

For brevity, we refer to the above steps as *f*, such that $T = f(P, V_k)$, where $V_k$ is the column matrix of the top *k* right singular vectors of *R*.

#### Target partitioning

The training procedure above involves computing the matrix $V_k^{T}V$, where $V_k$ is $k \times n$, which results in an $n \times n$ matrix. For large numbers of targets (*n*) the amount of storage required may exceed the amount of memory available and performance may be impacted. We use two strategies to ameliorate performance concerns. First, the randomized SVD algorithm of Halko et al. [16] offers several performance benefits compared to traditional decomposition methods. Second, we partition the *n* targets into disjoint sets of approximate size *j*, and treat each partition independently. *j* is a user-settable parameter, for the results shown here a value of 1,000 is used. Ad-hoc experiments have suggested that results are generally insensitive to the choice of *j* as long as $j>> 100$. The partitioning algorithm seeks to distribute partitions as uniformly as possible across targets. (Early experiments assigned many adjacent targets to a single partition and had poor sensitivity to CNVs that occupied a large percentage of the partition.)

Target partitioning greatly speeds the training procedure and reduces memory requirements from $O(n^2)$ to $O(j^2)$ without significantly impacting CNV calling accuracy.

#### Emission distribution parameter estimation

We assume all emission distributions are Gaussian with means and variances that differ across both states and targets, and we estimate the mean and variance for each state and target separately. Copy number states are described by the expected change in the raw read depth of the target - for instance, we assume a heterozygous deletion reduces raw read depth by approximately 50%, while a homozygous duplication increases raw read depth by 100%. Let vector *s* hold the coefficients describing the expected change in depth associated with each state. Under typical usage, we define 5 states and set $s = \{0.01, 0.5, 1.0, 1.5, 2.0\}$, where the elements correspond to homozygous deletion, heterozygous deletion, diploid, heterozygous duplication, and homozygous duplication or amplification. Special considerations are made for calls on the X and Y chromosomes in males, as described below.

To estimate the mean and variance parameters for target *t* and state *i*, we form ${\hat{D}}_{t,i}$ by multiplying column *t* of *D* by $s_i$. We then compute ${\widehat{T}}_{s,i} = f({\widehat{D}}_{s,i}, C)$, and take the sample mean and sample variance of column *t* of ${\widehat{T}}_{t,i}$ to be the mean and variance of the emission distribution for target *t* and state *i*. The above procedure is repeated for every target and copy number state and the resulting means and variances recorded for all states and targets. We refer to the full collection of parameter estimates and singular vectors *C* as a ‘model’, which is persisted and used to facilitate the CNV discovery procedure.

**Fig. 2:**
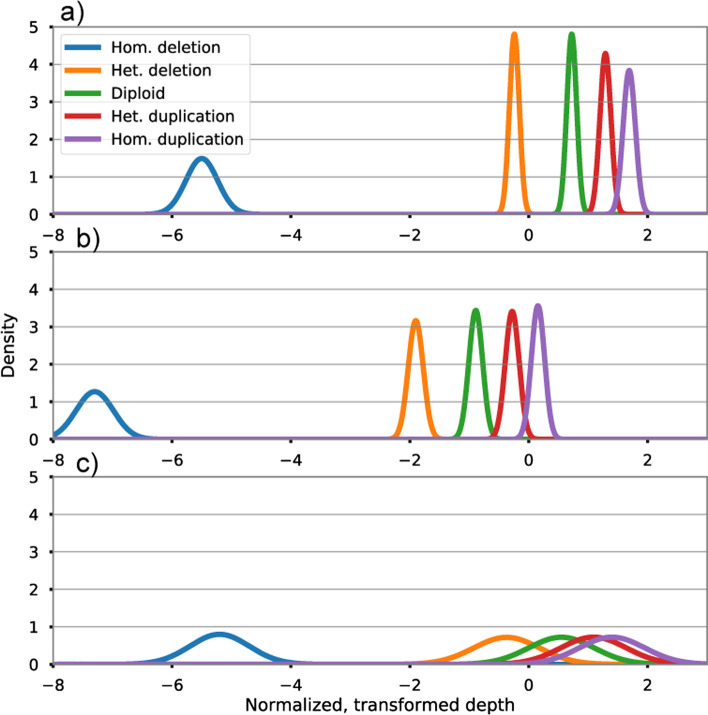
Example emission distributions illustrating between target variation. **a**, **b** Show well behaved targets with adequate separation between emission distributions, **c** demonstrates a low resolution target with substantial overlap between emission distributions

#### Target resolution calculation

Using the set of stored emission distribution parameters it is possible to calculate an ad-hoc value that reflects the power of a given target to resolve copy number status in general. For some targets emission distributions are well separated and have small variances relative to the difference in means between states (e.g. Fig. 2a), while at other targets the distributions may overlap substantially (Fig. 2c). CNV identification is likely to be impaired when the distributions overlap because posteriors for the states are also likely to overlap substantially. In some cases, it may be possible to detect certain CNV states, such as homozygous deletions, while resolution for other states may be relatively poor (Fig. 2c).

As a proxy for overall target resolution, we compute the Kullback–Leibler divergence [17] between the heterozygous deletion state and the diploid state. While the value is based only on the difference between two of the five typical states, we note that separation between the diploid and heterozygous deletion states is often closely correlated to separation between other states, and that the heterozygous deletion state is often the most relevant from a clinical standpoint. Our implementation can compute this statistic for every target in a saved model and emit the results in BED format. In practice, values less than approximately 10 appear to be associated with relatively poor CNV calling accuracy.

This procedure may be useful for understanding which targets are associated with poor resolution, and hence should be excluded from routine calling procedures. In a panel design context, such information might be used to generate formal statements regarding inclusion/exclusion of particular genes or exons.

#### CNV prediction

Prediction requires a set of parameter estimates and singular vectors as computed during training, and a set of sample depths taken over the same set of targets used for model training.

We construct a homogeneous Hidden Markov Model (HMM) with initial state vector $\pi = \{0.01, 0.01, 0.96, 0.01, 0.01\}$, a transition probability matrix *M*, and set of emission distribution parameters obtained from the stored model. We assume all emission distributions are Gaussian, with parameters estimed during training (see “Emission distribution parameter estimation” section). We construct a two-parameter variant of *M*, where one parameter describes the probability of moving ‘away’ from the diploid state and the other describes moving back ‘toward’ the diploid state. Specifically, we construct *M* as:

$$\begin{aligned} M = \left[ \begin{array}{ccccc} 1 - \beta &{} \beta &{} 0 &{} 0 &{} 0 \\ \alpha &{} 1-\alpha -\beta &{} \beta &{} 0 &{} 0\\ 0 &{} \alpha &{} 1-2\alpha &{} \alpha &{} 0 \\ 0 &{} 0 &{} \beta &{} 1-\alpha -\beta &{} \alpha \\ 0 &{} 0 &{} 0 &{} \beta &{} 1- \beta \\ \end{array}\right] \end{aligned}$$

where $\alpha$ and $\beta$ are set to 0.0025 by default. Smaller values of the $\alpha$ and $\beta$ favor fewer, larger CNVs, while larger values favor smaller, more numerous CNV calls.

Raw sample depths are transformed via *f* using singular vectors *C* obtained during training, and the transformed depths are treated as observations to obtain posterior state probabilities from the HMM using the Forward-Backward algorithm. Finally, we produce a list of most likely state probabilities using PMAP (pointwise maximum a posteriori, see [15]). Adjacent segments of identical most-likely state are joined into single regions, and all non-diploid regions are emitted as CNV calls.

### Comparison to other detection tools

We investigate the performance of our method on two different datasets, exomes and a large custom panel. The exome data was used for a simulation analysis, while the custom panel was used to explore accuracy in detecting CNVs previously discovered by array comparative genomic hybridization (aCGH) or multiplex ligation-dependent probe amplification (MLPA). Throughout we compare our method to Conifer [6], XHMM [7], Convading [8], ExomeDepth [18], Clamms [10], and Codex [12].

#### NGS data

50 exomes derived from whole blood were captured using the xGen Exome Research Panel v1.0 probe set from IDT. Exomes were sequenced on 4 separate runs of an Illumina HiSeq 4000 instrument to a mean read depth of approximately 150 reads. Reads were aligned to human reference genome GRCh37 with phiX and decoy sequences included using BWA MEM (v0.7.12, [19]). Potential PCR duplicates were identified and marked using Sambamba [20].

Read counting was performed using the methods recommended by each caller examined, often using either the DepthOfCoverage tool from GATK 3.7 [21] or the multicov facility in BEDTools [22], although ConVaDINg [8] implements read counting internally. For Cobalt, read counting was performed using the PySAM interface to samtools [23], and the total number of reads overlapping each CNV target (probe) was recorded.

In addition to the exome data, we analyzed 218 samples captured with a custom probe design manufactured by IDT. This large panel targets 4921 genes with 71,163 probes and has a footprint of 16 Mb. These samples were sequenced on 5 runs of a HiSeq 4000 instrument to a mean read depth of approximately 300. Data analysis was identical to that for the exomes.

#### Simulated CNV generation

Simulated CNVs were introduced into exome BAM files by either removing or duplicating existing reads. CNVs spanning 1, 3 or 10 exons were generated in separate BAM files, see Table 1. CNV locations were chosen uniformly from RefSeq alignments on the autosomes, and read counts were reduced by 50% to create heterozygous deletions and increased by 50% to create heterozygous duplications.

**Table 1:** Number of simulated CNVs by size

Number of exons	Number of CNVs
1	1000
3	300
10	100

## Results

We evaluate CNV caller accuracy with a combination of simulated CNVs and those detected by array CGH and confirmed with an orthogonal technology.

### Simulation analysis

Sensitivity and positive predictive value (PPV) were tabulated on a sample-by-sample basis given the optimal quality score cutoff for each caller and CNV size (see Table 2). A 50% reciprocal overlap (intersection-over-union/Jaccard) rule was used to determine if a simulated CNV was called correctly. Default sensitivity and specificity settings differed substantially across callers, preventing a simple comparison of raw CNV calls. To standardize caller output, we attempted to find the optimal quality score cutoff for each caller independently, then compared CNV callers using the caller-specific thresholds. For each caller candidate CNVs were called using high sensitivity settings and sensitivity/specificity curves were constructed using the caller-produced CNV call quality. The $F_1$ statistic was calculated at 10 different evenly-spaced quality values, and the quality score associated with the maximum $F_1$ score was chosen as the optimal quality threshold. One caller, Conifer, did not produce CNV quality scores and for this caller we did not perform quality threshold optimization.

**Table 2:** Quality thresholds used in simulation analysis

Caller	CNV size (exons)	Quality threshold
	1	0.95
Cobalt	3	0.95
	10	0.96
	1	5
XHMM	3	10
	10	8
	1	− 6.4
ExomeDepth	3	64
	10	12
	1	0
Convading	3	0
	10	0.3
	1	0.85
Clamms	3	0.95
	10	0.93
Conifer	1–10	NA
	1	0.52
Codex	3	30
	10	118

**Fig. 3:**
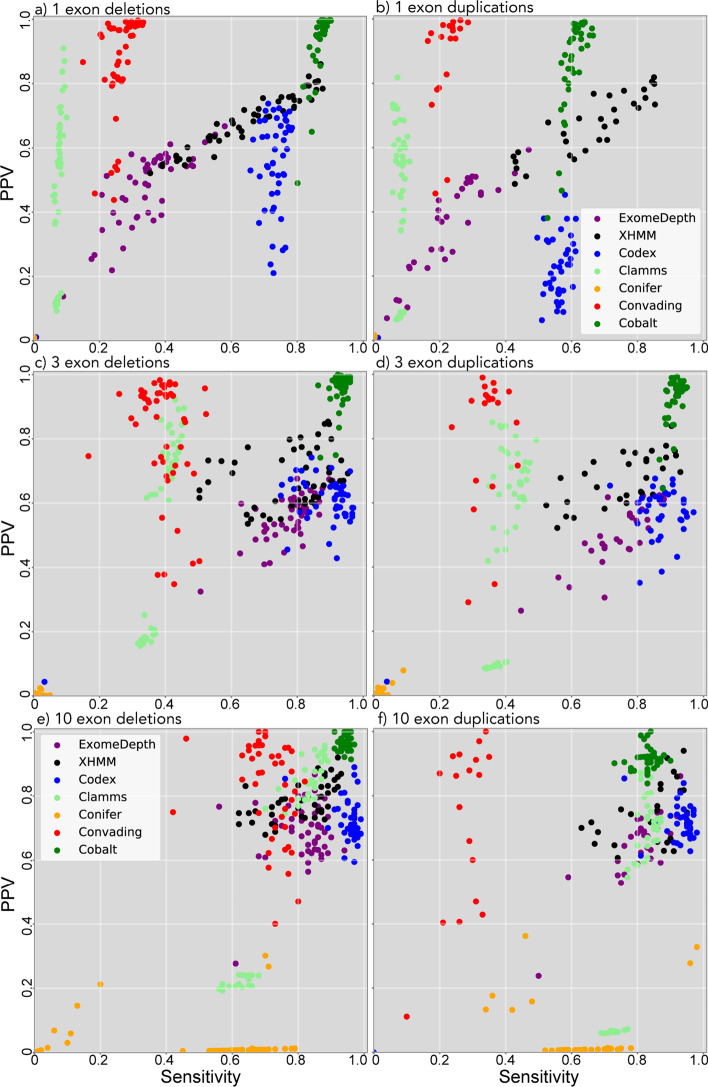
Per-sample sensitivity and positive predictive value (PPV) for simulated deletions (left column) and duplications (right column) CNVs spanning 1 (top row), 3 (middle row), and 10 (bottom row) capture targets

For deletions in all size categories Cobalt demonstrated consistently high sensitivity and PPV, and achieved the highest sensitivity and PPV among all callers for deletions spanning 1–3 exons (Fig. 3a, c). For deletions spanning 10 exons sensitivity was slightly higher for CODEX [12], though at the cost of significantly lower PPV (Fig. 1e). For duplications spanning 1–3 exons Cobalt demonstrated the highest mean sensitivity and PPV of the callers we examined (Fig. 3b, d), although overall sensitivity for 1-exon duplications was low (61%). For 10-exon duplications, CODEX again achieved higher sensitivity (91% compared to 83%) but significantly lower PPV (70% vs 92%, Fig. 3f).

### Previously detected CNVs

We examined 68 samples containing CNVs previously detected by aCGH or MLPA in addition to 160 ‘background’ samples which had not undergone any CNV detection procedure. Unlike the simulation analysis, samples were separated by sex, yielding 78 male and 82 female background samples. Sequencing and primary analysis were performed as described in the “NGS data” section. To determine if a true CNV was detected, we computed a modified Jaccard (intersection-over-union) statistic. Specifically, we computed the intersection of CNV targets in the true CNV and the CNV targets overlapped by CNV calls, and compared this to the union of true CNV targets and the called CNV targets. If no called CNVs overlapped the true CNV a value of 0 was recorded. True CNVs with a Jaccard value of 0.5 or greater were labeled as correctly called. Caller specific quality thresholds were used as given in Table 2.

**Table 3:** Number of previously detected CNVs

Number of CNV targets	CNV type	Number of CNVs
1–4	Deletion	19
5–9	Deletion	14
10+	Deletion	5
1–4	Duplication	9
5–9	Duplication	3
10+	Duplication	12

**Fig. 4:**
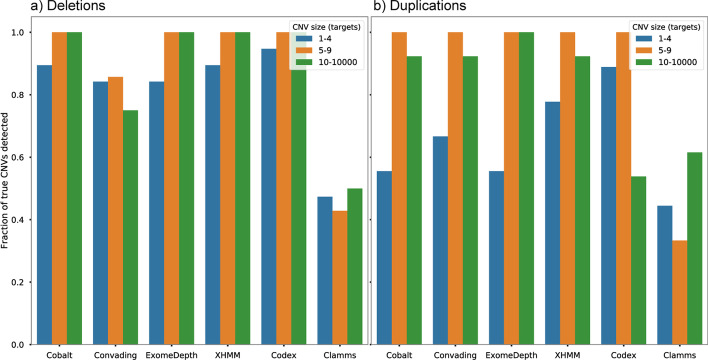
Sensitivity of Cobalt and other CNV detection tools on **a** deletion and **b** duplication CNVs of different sizes

Cobalt demonstrated a sensitivity comparable to other top-performing callers with values near 90% for small (1–4 target) deletions and near 100% for medium to large deletions (spanning 5 or more targets). For duplications, Cobalt struggled to detect small (1–4 target) CNVs and yielded a sensitivity of near 50%, but correctly detected 90–100% of medium and large duplication CNVs. CODEX achieved similar sensitivity levels for deletions and substantially higher sensitivity to small duplications, but relatively low sensitivity to large (10 or more target) duplications.

The total number of CNV calls varied substantially across callers (Fig. 3). While the true status of these calls is unknown, we suggest that the total number of CNV calls is positively correlated with the false discovery rate of the caller for the following reasons. First, if our sensitivity data (Figs. 3, 4) are accurate, then approximately 90–100% of true CNVs are detected, thus large discrepancies in the number of CNV calls made in total are more easily explained by additional false positive calls rather than very large numbers of true, previously undetected CNVs. Second, prior analysis with aCGH has suggested that a typical number of CNVs per sample is between 0–10, and that very few samples contain hundreds (or thousands) of CNV calls.

**Fig. 5:**
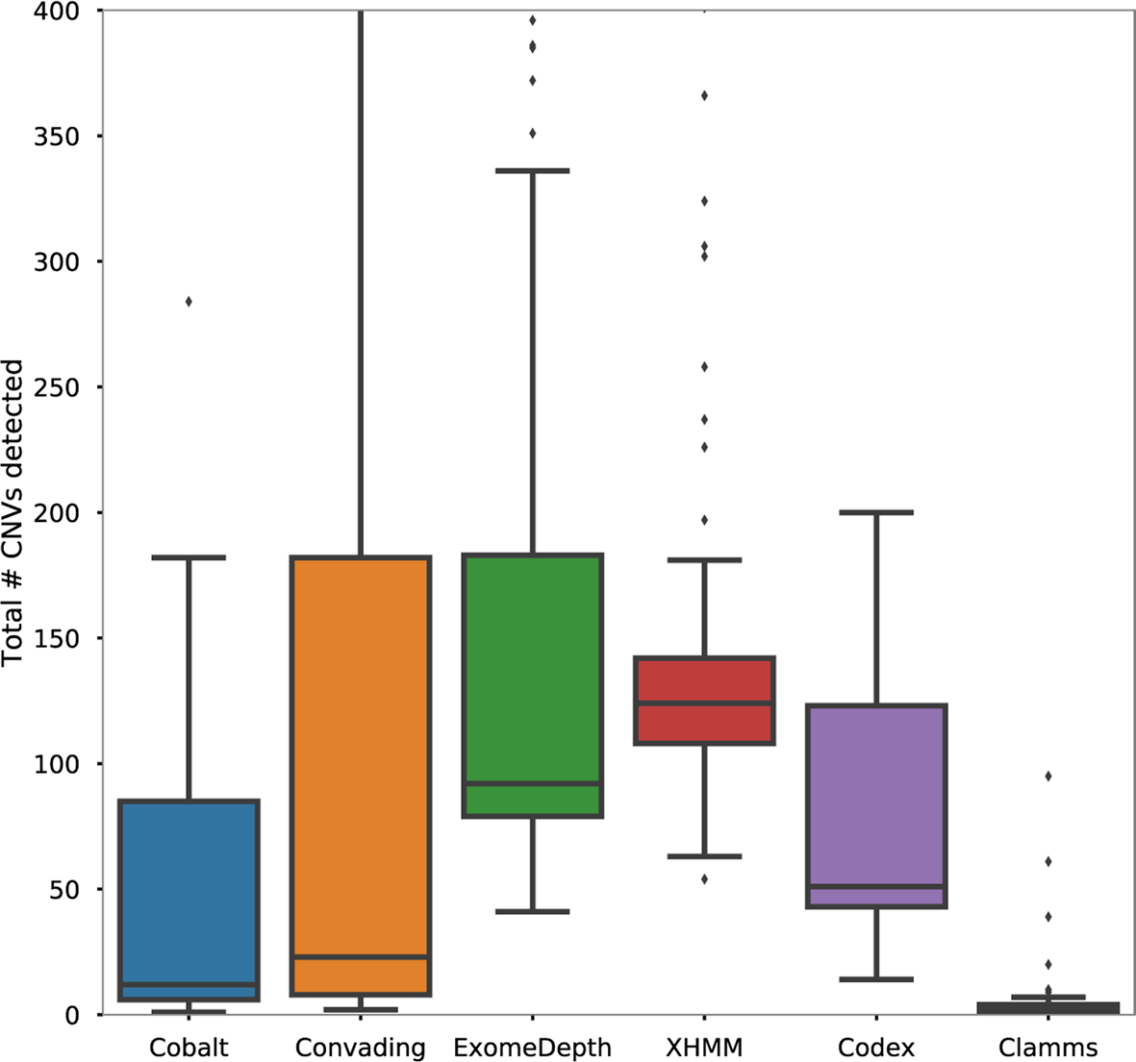
Total number of CNVs, including both true and false positive calls, detected by Cobalt and other callers in samples with previously detected CNVs

If the total number of CNV calls is correlated with false discovery rate (FDR), then Cobalt achieves the second-lowest FDR of all callers (Fig. 5) with a median number of 12 CNV calls per sample. The other callers that achieved similar sensitivity, CODEX, ExomeDepth, and XHMM, discovered 51, 92, and 124 calls per sample. Only Clamms yields fewer CNV calls per sample (median 2), although Clamms also demonstrated low sensitivity overall with values less than 50% for most CNV classes.

## Discussion

The Cobalt CNV caller introduces several improvements over previous techniques that improve specificity while maintaining high sensitivity. Most importantly, Cobalt uses a unique method to generate accurate, target-specific parameters for the HMM. For each copy-number state at each target, these parameter estimates are produced by modifying the background sample depths to produce a pseudo-CNV, then capturing the mean and variance of the background depths after log-normalization and removal of singular vectors. For comparison, the HMM of Fromer et al. [7] assumed that the means and variances of the emission distributions were constant across all targets.

An additional refinement is use of the PMAP (pointwise maximum a posteriori) criterion when decoding the HMM. Most previous HMM-based CNV callers employ the Viterbi algorithm to assign copy-number states to targets. While Viterbi yields the single most likely path through states across targets, it does not necessarily maximize the number of correctly called targets, while PMAP does [24]. One drawback to PMAP is that it may yield *inadmissible* paths that have 0 prior or posterior probability. For instance, it may be the case that PMAP indicates a duplication target adjacent to a deletion target, even though the transition matrix specifies 0 probability for such a transition. While unsatisfying, we appeal to the approximate nature of the HMM transition matrix and note that few real-world cases justify entries of exactly 0 in the transition matrix. While it is possible to construct a PMAP decoder to yield only admissible paths [15], we leave this refinement along with exploration of more sophisticated decoding strategies to future work.

When used to predict simulated CNVs in exome data, Cobalt achieved consistently high sensitivity and PPV (Fig. 3), with the exception of single exon duplications. The Codex algorithm [12] demonstrated somewhat higher sensitivity for several categories, in particular for large duplications, but the improved detection rate comes at the cost of substantially lower PPV. Results for previously detected CNVs were similar, although resolution was somewhat impaired by the smaller number of true CNVs available. Cobalt yielded sensitivity indistinguishable from other top-performing callers but made far fewer CNV calls overall (Figs. 4, 5), strongly suggesting that Cobalt yields simultaneously high sensitivity and PPV.

PPV is particularly important in the clinical laboratory setting for several reasons. Orthogonal confirmation of putative CNV calls is often expensive and time-consuming; in fact, a primary motivation for calling CNVs from NGS data is to avoid the cost and complexity of running array-based CNV detection in addition to NGS on every sample. In the absence of routine orthogonal confirmation, labs seeking to avoid erroneous patient results labs must optimize for both sensitivity and PPV. In this setting, Cobalt may represent an appealing choice because it offers sensitivity similar to other high performing callers while making significantly fewer calls overall.

Generally speaking, comparisons of CNV detection tools are beset by many difficulties. First, caller performance is likely to vary substantially with features of the input data, including depth, number of targets, and the structure of variability, especially batch variability. Some callers may perform well in the face of substantial variability, while others might excel only with minimally variable data. Similarly, relative caller accuracy may shift with mean read depth. An additional factor is the amount of parameter optimization performed for each caller. In this study we have performed only minimal optimization and have relied instead on identification of optimal quality score thresholds to normalize results across callers. Nonetheless, some callers may have significantly improved relative accuracy with careful tuning of parameters.

## Conclusions

Cobalt is a software tool to detect germline copy-number variants from hybrid-capture NGS data. By tuning emission distribution parameters individually for each target and decoding the HMM with the PMAP algorithm, instead of the typical Viterbi decoder, Cobalt maintains high sensitivity while detecting significantly fewer false positive calls than other detection tools. Cobalt is freely available as an open source project under the permissive GPL (v3) license.

## Availablility and Requirements

Project name: Cobalt

Project home page: https://github.com/ARUP-NGS/cobalt

Programming language: Python 3

Requirements: Python version 3.6 or greater

License: GNU General Public License v3.0

Restrictions for non-academic use: None.

## Data Availability

Source code available at https://github.com/ARUP-NGS/cobalt. The datasets generated and analysed during the current study are available in that repository under the data/ directory (https://github.com/ARUP-NGS/cobalt/data/).

## Abbreviations

NGS: Next generation sequencing
CNV: Copy number variant
HMM: Hidden Markov model
PPV: Positive predictive value
WES: Whole exome sequencing
BED: Browser extensible data
PMAP: Pointwise maximum a posteriori
